# Exploration of the affordances of mobile devices in integrating theory and clinical practice in an undergraduate nursing programme

**DOI:** 10.4102/curationis.v38i2.1510

**Published:** 2015-09-28

**Authors:** Juliana J. Willemse, Vivienne Bozalek

**Affiliations:** 1 School of Nursing, University of the Western Cape, South Africa; 2 Director of Teaching and Learning, University of the Western Cape, South Africa

## Abstract

**Background:**

Promoting the quality and effectiveness of nursing education is an important factor, given the increased demand for nursing professionals. It is important to establish learning environments that provide personalised guidance and feedback to students about their practical skills and application of their theoretical knowledge.

**Objective:**

To explore and describe the knowledge and points of view of students and educators about introduction of new technologies into an undergraduate nursing programme.

**Method:**

The qualitative design used Tesch’s (1990) steps of descriptive data analysis to complete thematic analysis of the data collected in focus group discussions (FGDs) and individual interviews to identify themes.

**Results:**

Themes identified from the students’ FGDs and individual interviews included: mobile devices as a communication tool; email, WhatsApp and Facebook as methods of communication; WhatsApp as a method of communication; nurses as role-models in the clinical setting; setting personal boundaries; and impact of mobile devices in clinical practice on professionalism. Themes identified from the FGD, individual interviews and a discussion session held with educators included: peer learning via mobile devices; email, WhatsApp and Facebook as methods of communication; the mobile device as a positive learning method; students need practical guidance; and ethical concerns in clinical facilities about Internet access and use of mobile devices.

**Conclusion:**

The research project established an understanding of the knowledge and points of view of students and educators regarding introduction of new technologies into an undergraduate nursing programme with the aim of enhancing integration of theory and clinical practice through use of mobile devices.

## Introduction

The global increase in and availability and affordability of mobile devices has made them indispensable in day-to-day social networking. Such mobile devices are embraced by young people, giving them a sense of ownership whilst engaging with the devices (Pachler, Bachmair & Cook 2010:3). There has also been an increase in the use of mobile technology to enhance teaching and learning practices (Rambe & Bere 2013; Sharples et al. 2013). Mobile learning (m-learning) has become a new trend in the education sector, with an exponential acceleration in the variety of applications afforded by mobile devices (Aharony 2014:1; Gupta & Koo 2010:75).

The American Association of Colleges of Nursing, the National League for Nursing and the Institute of Medicine, major forces in professional health care and nursing education, advocate incorporation of mobile devices in nursing education for integration of theory and practice (George et al. 2010). Handheld technological devices or PDAs (personal digital assistants) were effectively incorporated into nursing education programmes and provided students with a rich resource of reference material that was available and up to date. Almost 80% of students who participated in this study indicated that they successfully used PDAs as an educational resource in both the classroom and the clinical environment (George et al. 2010:371).

M-learning is fundamentally defined as ‘learning with mobile devices’ and has the potential to extend the philosophies of learning through innovation (Gupta & Koo 2010:75). Rushby (2012:355) specifies that research about m-learning can be divided into four areas: pedagogy, administrative issues and technological challenges, ensuring sustainable development in education using m-learning, and the impact of new applications. Thus the possibility of introducing ‘learning with mobile devices’ to assist students in bridging the gap between theory and clinical practice motivated the need to explore introduction of mobile devices as an emerging technology in the undergraduate nursing programme at a higher education institution (HEI) in the Western Cape Province, South Africa.

In a study by Willemse, Jooste and Bozalek (2014:195) third-year undergraduate nursing students and educators participated in a quantitative survey at an HEI in the Western Cape Province. The survey explored and described the perceptions of participants on the potential use of mobile devices in relation to integration of the theory and clinical practice in primary health care (PHC) through m-learning. The study found that 79.8% of the student population were using mobile devices on a daily basis.

Students identified the following use of mobile applications on their mobile devices: 9.5% for email, 79.8% for WhatsApp and 75% for Facebook. However, it was interesting that when asked which application was the most suitable for receiving tasks from their educators related to coursework, 76.2% indicated email, 64.3% WhatsApp and 46.4% Facebook. The difference in the results between the two questions identified the need for clarification and deeper exploration of some of the results from the survey. The need to conduct a qualitative study, for example through focus group discussions (FGDs) and individual interviews supported by observational notes, could refine and extend the general picture developed from the quantitative survey completed.

### Problem statement

A concern emerged amongst educators facilitating a PHC module at an HEI in the Western Cape, South Africa, that their undergraduate nursing students seemed unable to integrate their theory with their clinical practice. It was identified that students refrained from carrying much-needed resource material that could assist them with this integration through research (such as clinical module guides and textbooks) into their clinical environment. Educators agreed that innovative measures had to be researched to assist students with this integrative process, to ensure their continued academic and clinical growth leading to success. The introduction of mobile devices was identified, as it had the potential to become a resource which could assist students to address the problem of needing to have resource material available at any time or in any given place, and to be able to do research on conditions or procedures whilst off campus and in the clinical field (Cook, Pachler & Bradley 2008:3).

#### Aim of the study

The aim of the study was to explore and describe the affordances of mobile devices to integrate theory and clinical practice in an undergraduate nursing programme at an HEI in the Western Cape.

#### Research objectives

The research objectives of this study were to:

- Explore and describe the knowledge and points of view of students and educators about introduction of new technologies into an undergraduate nursing programme to enhance integration of theory and clinical practice using mobile devices.
- Identify the affordances of mobile devices with the introduction of new technologies into an undergraduate nursing programme to enhance integration of theory and clinical practice using mobile devices.

### Definition of concepts

**Higher education institution:** The *Higher* *Education* *Act* , 1997, as amended in 2010, states as follows:

A ‘Higher Education Institution’ means any institution that provides higher education on a full-time, part-time or distance basis and which is –

*(a)* Merged, established or deemed to be established as a public HEI under this Act;
*(b)* Declared as a public HEI under this Act; or
*(c)* Registered or provisionally registered as a private HEI under this Act.

**Mobile learning (m-learning):** Koole (2009), as cited in Kenny et al. (2009:77), sees m-learning as resulting from the combination of mobile technologies, the capacity of human learning, social communication, and interaction with the device, the learner and the societal aspects of learning.Herrington et al. (2009:2) argue that in spite of the potential use of m-learning in higher education, it appears to be a mostly instructive, teacher-centred paradigm rather than a more constructivist paradigm.

**Mobile devices:** Mobile devices are associated with small wireless, portable, handheld devices, such as cellular phones, smart phones, PDAs, MP3 players, portable game devices, handhelds, tablets, notebooks and laptops (Kukulska-Hulme & Traxler 2005:2; Traxler 2007:1–12; Wagner 2005:40–53).

### Significance of the study

It is anticipated that the study would contribute to implementation of an original intervention which incorporates mobile devices and m-learning, based on the results of the exploration and description of its affordances into a programme to integrate theory and clinical practice in an undergraduate nursing programme at an HEI in the Western Cape.

## Theoretical framework: Affordances of mobile technology

Salomon (1993), as cited in Conole and Dyke (2004), refers to ‘affordances’ as the perceived and actual properties of an object, primarily the functional properties, that determine just what and how the object could possibly be used. Gibson (1979:127) and Bower (2008:5) provide the following first definition of ‘affordances’:

The affordances of the environment are what it offers the animal, what it provides or furnishes, either for good or ill. The verb to afford is found in the dictionary, but the noun affordance is not. I have made it up. I mean by it something that refers to both the environment and the animal in a way that no existing term does. It implies the complementarity of the animal and the environment.

Bower (2008:3) matches teaching and learning tasks with appropriate learning technologies by looking at the action potential of the technology. The dynamic model of Bower (2008:3) enables a better understanding of how teachers identify different kinds of knowledge as valuable in an attempt to support students’ ability to learn content knowledge. The categories of affordances of mobile technology provided a framework for the data analysis in this exploration of the affordances of mobile devices in integrating theory and clinical practice in an undergraduate nursing programme.

The affordance framework presented in Table 1 defines not only technological affordances, but includes social and educational affordances.

**TABLE 1:** Classification of affordances (adapted from Bower 2008:6).

Number	Categories of affordances	Action possibilities
1	Media affordances	Readability, writability, viewability, drawability, listenability, speakability, watchability, video-production-ability
2	Spatial affordances	Resizeability, movability
3	Temporal affordances	Accessibility, recordability, playbackability, synchronicity
4	Navigation affordances	Browsability, linkability, searchability, data-manipulation-ability
5	Emphasis affordances	Highlightability, focusability
6	Synthesis affordances	Combinability, integratability
7	Access-control affordances	Permission-ability, shareability

*Source*: Adapted from Bower, M., 2008, ‘Affordance analysis - Matching learning tasks with learning technologies’,* Educational Media International* 45(1), 3–15

## Research method and design

The study had a qualitative, exploratory, descriptive and contextual design which provided individual rich data about the phenomena, environment, interactions, meaning and everyday experiences of the participants (Rubin & Babbie 2011:134). The qualitative design explored and describe the knowledge and points of view of students and educators on the affordances of mobile devices to enhance integration of theory and clinical practice in an undergraduate nursing programme.The qualitative data collection methods provided an in-depth understanding through FGDs and individual interviews on the nature of the knowledge and points of view of students and educators on the above topic.

### Research setting

The research study was conducted in the context of an undergraduate nursing programme at an HEI in the Western Cape Province.

### Research population and sample

The population of this study included an ‘accessible’ population, since the researcher had reasonable access to them (Burns & Grove 2005:342; Teddlie & Tashakkori 2009:170). The participants included all third-year undergraduate nursing students (*n* = 100) registered for the PHC module, a semester module at a university, and their educators (*n* = 5) who facilitated that module. All members of the accessible population who had volunteered to take part in the study were included in the sample (Hek, Judd & Moule 2003:67). The purposive or selective sampling, a type of non-probability sampling, was a conscious selection by the researcher to include all participants in the study who represented the phenomena being studied (Patton 2002:44).

The accessible population served as the sample (Creswell & Plano Clark 2011:174). To be included in the study the students and educators had to comply with specific characteristics (Burns & Grove 2001:376) and had to be:

- in possession of a personal mobile device, unless the researcher provided a specific device for the purposes of the study;
- prepared to use their personal mobile devices as a tool to take part in the study; and
- registered for the PHC module (students) or an educator who facilitated the module.

In total four FGDs and eight semi-structured face-to-face in-depth individual interviews were held with students, whilst one FGD, one discussion group and three semi-structured face-to-face in-depth individual interviews were held with educators. Initially six FGDs were planned, three from each of the two classes registered for the PHC module, but individuals from both classes withdrew from the study, leaving only enough students for four sessions, two from each class. A total of 10 semi-structured face-to-face in-depth individual interviews were planned with student participants, but after carrying out eight with students and 3 with educators no new or relevant data emerged, and it was evident that theoretical saturation had been reached. This implies that no new or relevant data emerged in the themes or sub-themes and is representative in terms of its properties and to the extent that validation can be confirmed and the relationship between the themes or sub-themes are well established and confirmed (Bryman 2012:421; Kumar 2005:68; Strauss & Corbin 1998:212).

### Data collection

Data were collected during FGDs and individual interviews with students, and an FGD, individual interviews, and a discussion session with educators. In the FGD participants are encouraged, in a conducive environment, to share their viewpoints related to the researcher’s specific area of interest. FGDs usually include no more than 6–10 participants, which allows everyone in the group an opportunity to participate. The size of the group would depend on how much time is needed to start the discussion, and to ensure that every member is awarded an opportunity to respond (De Vos et al. 2011:360). Focus group sessions were conducted with groups of six participants per group.

An individual interview is a data collection method where the researcher conducts interviews with one person at a time (Plano Clark & Creswell 2010:258). These were conducted to enable participants to provide more detail on issues raised during the FGDs. This also provided participants who were not comfortable to share their thoughts within the focus group setting to share their thoughts in a comfortable environment (Plano Clark & Creswell 2010:258).

The transcripts of the FGDs and individual interviews held with students and educators, including the discussion session held with educators, were collated for each participant; this provided the researcher with a ‘sense’ of the text. Participants were invited to review their individual transcripts to confirm the correctness of the transcripts before the data analysis process commenced.

### Data analysis

Without manipulating the data obtained, they were thematically analysed and categorised into themes in an attempt to collate an accurate exploration and description with regard to the research problem identified (Cook et al. 2008:4). Main themes were identified from the students’ FGDs and individual interviews and the FGD, individual interviews, and a discussion session held with educators, as presented in Table 2.

**TABLE 2:** Themes identified.

Themes identified by students	Themes identified by educators
Mobile devices as a communication tool	Peer learning via mobile devices
Email, WhatsApp and Facebook as methods of communication	Email, WhatsApp and Facebook as methods of communication
WhatsApp as a method of communication	Mobile devices as a positive learning method
Nurses as role-models in the clinical setting	Students’ needs for practical guidance
Setting personal boundaries	Concerns about Internet access
Mobile device impact on professionalism in clinical practice	Mobile devices and ethical concerns at facilities

The discussion on the findings of this study was guided by assigning affordances to each of the findings with the purpose of creating a meaningful theoretical framework.

## Findings and discussion

The themes identified from the thematic analysis of the FGDs and individual interviews concluded with students who participated in the study are henceforth summarised.

## Mobile devices as a communication tool

Participants acknowledged that the use of mobile devices made communication with their educators easier, but emphasised that a limited number of students were using smart phones. The researcher recognised that some students were not able to download WhatsApp or Facebook due to the model of their mobile device not affording this functionality or application:

‘We are here, we’re talking about cell phones that we can access WhatsApp and Facebook. But I’m sure that not all of us here at school have smart phones.’ (Participant 3, FGD 4)

The suggestion was raised that the study should focus on the short messaging system (SMS), but it was discussed that an SMS might not support group interactivity or group discussions. The participants did not seem to be aware that SMSs are in fact flexible and messages could be sent to either individual students or groups of students. The challenge with using an SMS would be the costs that might be incurred for the students and educators (Idrus & Ismail 2010:2768). A participant found the mobile device had linkability to with lecturers: ‘For me, a mobile device makes it easier for the communication part … with your clinical supervisors and lecturers [*educators* ] and for learning and studying’ (Participant 5; individual interview). However, participants acknowledged that they had access to the functionalities of mobile phone applications that included Facebook and WhatsApp. Due to the time delay for some students to access their email accounts, one suggested that students should be encouraged to request their parents to purchase them a mobile device that could access WhatsApp to ensure that they would not miss out on the ‘ongoing communication’:

‘I think, probably if we can encourage the student maybe to ask their parents to buy them phones so that they can be able to download WhatsApp and communicate.’ (Participant 6; FGD 3)

### Email, WhatsApp and Facebook as methods of communication

The affordances of email, WhatsApp and Facebook include readability, viewability, writability, accessibility, browsability, linkability and shareability, whilst WhatsApp and Facebook also include listenability and watchability (Bower 2008:12). The affordances of WhatsApp and Facebook allow students and educators to discuss issues in ‘real time’ and they are able to share course-related information when in the clinical environment. A depiction of the affordances of email, WhatsApp and Facebook identified is presented in Table 3.

**TABLE 3:** Affordances identified – email, WhatsApp and Facebook.

Technological applications	Affordances																							
Read­ability	View­ability	Listen­ability	Watch­ability	Writ­ability	Draw­ability	Speak­ability	Mov­ability	Video-production-ability	Resize­ability	Play­backability	Accessi­bility	Record­ability	Synchronous-ability	Brows­ability	Search­ability	Data-manipulation-ability	Link­ability	Highlight­ability	Focus­ability	Combin­ability	Integrat­ability	Permission-ability	Share­ability
Email	√	√			√							√			√		√	√					√	√
WhatsApp	√	√	√	√	√		√	√	√		√	√	√		√	√	√	√					√	√
Facebook	√	√	√	√	√			√			√	√			√	√		√					√	√

Participants voiced their opinion that: (1) email was their preferred method of communication, followed by (2) WhatsApp and (3) Facebook. Participants confirmed that email should be the preferred communication application, since it was used by many and could accommodate larger documents, but mentioned that some individuals might experience challenges with the use of emails:

‘And email specifically because it can accommodate larger documents that cannot be accommodated on WhatsApp.’ (Participant 4, FGD 1)

‘… for me I think its email being the first one … that will be most usable by many people … but some people, they struggle to … I think to use … to use email somehow.’ (Participant 3, FGD 1)

Students participating in the study decided to include WhatsApp as a method of communication: ‘WhatsApp is the most used under [*sic* ] students at the moment’ (Participant 1; FGD 1). Participants clarified amongst themselves that both WhatsApp and emails should be used to communicate ‘as a good option’ due to accessibility and affordability: ‘I think we can use WhatsApp or the social networks, even the emails during our time, breaks, and after work; not during the work time’ (Participant 4, FGD 3).

Facebook was also indicated as one of the preferred methods of communication, though to a lesser extent than email and WhatsApp. Ivala and Gachago (2012:163) recommend that the daily use of technology, including Facebook and blogs, by students at HEIs could promote student engagement and communication and could lead to improved performance and retention of students. Two students commented as follows: ‘And also Facebook … it’s the other … method of communication’ (Participant 2, FGD 1), ‘Choose WhatsApp and email … for me Facebook is a social network and for me I would not want the friends I have on Facebook to see my studies’ (Participant 1, FGD 1).

In terms of the affordability of email, WhatsApp and Facebook, WhatsApp offers the most affordances of a mobile technological intervention.

#### WhatsApp as a method of communication

Students participating in the study decided that mobile devices with access to the social media application WhatsApp could be used to guide clinical practice by, for example, streaming short instructional videos and asking lecturers questions. More recent updates have enhanced the functionality of WhatsApp as an application, making it more user-friendly. WhatsApp was created by Brian Action and Jan Koum in 2009 to improve communication and make the distribution of multimedia messaging easier and faster. The purpose of WhatsApp was to replace SMSes with an Internet-based platform that provides unlimited text whilst evading or escaping the international fees that mobile providers charge (Yeboah & Ewur 2014:157). Some of the students’ comments on WhatsApp:

‘If you’re using WhatsApp, you can easily ask the questions to your lecturers, if you’re busy with an assessment.’ (Participant 1, FGD 3)

‘Because it is instant … I can quickly ask you, this is what the patient came with. This is my thought on what might be the problem or query or whatever.’ (Participant 2, individual interview)

‘I think WhatsApp would be the most appropriate one. Because with WhatsApp, if you don’t have airtime like my phone, if I have R2 on my phone I cannot make a call, I cannot send an SMS, but I can still WhatsApp.’ (Participant 1, FGD 4)

Currently WhatsApp can be regarded as the cross-platform between the instant messaging application and mobile instant messaging (MIM) on smart phones (Church & De Oliveira 2013:352). The study participants of Church and De Oliveira (2013:354) indicated that they sent more messages using WhatsApp compared to SMS, since they were not limited in terms of number of characters and content format on WhatsApp. Participants in this study also perceived their WhatsApp conversations as natural and conversational in nature, with the writing and receiving of messages that provide the sense of an open conversation, as if one was actually talking to a person.

The exploration and points of view of students indicated that WhatsApp offered the following affordances (Table 4) which could contribute to the introduction of new technologies for use on mobile devices in an undergraduate nursing programme to enhance integration of theory and clinical practice.

**TABLE 4:** Application the students felt had the most affordances – WhatsApp.

Technological applications	Affordances																							
Read­ability	View­ability	Listen­ability	Watch­ability	Writ­ability	Draw­ability	Speak­ability	Mov­ability	Video-production-ability	Resize­ability	Play­backability	Accessi­bility	Record­ability	Synchronous-ability	Brows­ability	Search­ability	Data-manipulation-ability	Link­ability	Highlight­ability	Focus­ability	Combin­ability	Integrat­ability	Permission-ability	Share­ability
WhatsApp	√	√	√	√	√	-	√	√	√	-	√	√	√	-	√	√	√	√	-	-	-	-	√	√

### Setting personal boundaries

Participants provided varied responses about the personal boundaries of lecturers and clinical facilitators in a WhatsApp group. One participant, felt that it would be overstepping boundaries to have the cell (mobile) phone number of a lecturer, due to the perception that students and lecturers ‘don’t communicate often via WhatsApp or BBM or whatever’ (Participant 1, FGD 1). That might also impact on students sending a message outside of the time arranged for communication and discussion:

‘I was thinking more like, if for instance, she has most probably children and all of that, and she also has a life. Because I know some of the lecturers are still studying as well. So, we can’t impose on their lives. So let’s use each other as a resource.’ (Participant 1, FGD 1)

The recent dramatic uptake of social networking in education provides an open door for learning, but it could lead to potential challenges. Ethical integrity of both the student and educator is of utmost importance, as is respect for personal boundaries (Aragon *et al.* 2014:25).

### Nurses as role-models in the clinical setting

A participant remarked that their observation of what nurses did in clinical practice was what they would essentially duplicate or replicate as best practice, since they viewed those nurses as their role-models:

‘Because basically, the nurses [*in clinical practice* ] are like our little videos that we watch, because this is what we literally do … we stare at them … they’re holding it like that, so I must hold it like that … That’s why we tend to do the things that they do. Knowing it’s not the right way, but hey, it’s how I was taught.’ (Participant 5, FGD 2)

Nursing practice requires nurses to assist patients in an attempt to contribute to the health or recovery of the patients in terms of being able to perform unaided actions with the necessary strength, will and knowledge. The practice involves provision of physical and emotional support to the sick, helpless, and wounded (Freshwater & Maslin-Prothero 2005:401; Harris, Nagy & Vardaxis 2009:1301).

### Impact on professionalism of mobile device use in clinical practice

Participants were concerned about the possible negative impact that mobile device use might have on professionalism in the clinical environment:

‘What about the professionalism? If you’re ‘WhatsApping’ during the time of work and everything and the patient is there. They would think you’re not caring or anything … I think it would also affect the professionalism of nursing.’ (Participant 4, FGD 3)

This participant considered it important to leave the patient in the care of another healthcare provider and to be excused from the consultation room to communicate via a mobile device with the clinical facilitator with the purpose of seeking answers or guidance to questions that arose:

‘I think it would help in a case where you need answers now. Then you excuse yourself then at least you leave that person with someone else to help.’ (Participant 4, FGD 3)

A participant in a study by Russel, Gentzler and Wood (2014:216) experienced difficulties with changing the culture from traditional record-keeping to the use of mobile technology. Some nurses on duty found using a mobile device difficult.

The researcher also summarised the themes identified during the thematic analysis of the FGD, individual interviews and discussion session concluded with educators who participated in the study, as outlined below.

#### Peer learning via mobile devices

Educators were adamant that the student groups could facilitate peer group discussions that would create a sense of learning from one another:

‘I’m also thinking, if we set up groups then they can actually … like peer guidance, they can actually advise each other. Sometimes they are more comfortable speaking to a fellow student.’ (Educator 3, FGD)

Kukulska-Hulme (2010:181) propagates the efficient learning and communication tools of mobile technology by a wider range of students in diverse settings. A research study with secondary school learners shows that their mobile devices afford them mobility to work on an activity in different settings. Participants adopted a notebook computer for the study and they assisted one another when technical problems arose; this could be regarded as a mobile social support group (Gaved et al. 2010:187).

### Email, WhatsApp and Facebook as methods of communication

Initially there were varied responses to ascertain which method/s of communication would be most suitable for students for augmenting integration of theory and clinical practice. The strongest responses from educators in this regard were the use of (1) email, followed by (2) WhatsApp and (3) Facebook; they ranked these applications in the same order that the student participants did. The affordances thus remained as discussed previously (Table 3).

Educators advised that emails should be used as a communication platform to disseminate videos and larger documents to students: ‘We can always put attachments on the email or short videos or slides about physiology, especially when it comes to the ears’ (Educator 1, individual interview).

The educators identified that WhatsApp could be used as a learning platform, but that students had to ensure that they transferred all prior knowledge to the platform. Students could be probed with a picture or a case-based scenario in the WhatsApp group to assist them with self-directed learning and revision. Students could even use the platform to obtain clarity about issues discussed in class, especially those too scared or shy to ask questions in class:

‘We can use the mobile device as an asset for teaching and learning, because we can post on, I use WhatsApp …’ (Educator 1, individual interview)

‘So, they can send in their queries or anything that they find in the clinic … and you can have a discussion over WhatsApp, which is applicable to what they have found in the clinic.’ (Educator 2, FGD)

One educator, however, identified a limitation to using WhatsApp, that is when a student did not have access to an Internet connection.

One of the participants shared an experience of a student who was a friend on their Facebook profile:

‘I have one of the current students is [*sic* ] on my Facebook account and … the student were [*sic* ] so excited for the first time she posted a status on Facebook, she saw the tympanic membrane for the first time … I think that was an opportunity whereby we could have … I don’t want to say, test her knowledge, but to expand. But what did you see about the tympanic membrane? How did it look? At the same time, her other friends, her colleagues could have learnt from that opportunity.’ (Educator 4, FGD)

‘Educators indicated that they would support an intervention and regarded it as appropriate to integrate theory and clinical practice by using WhatsApp and email in conjunction with each other as a mode of communication with students: “They can communicate to us via for example, emails or the WhatsApp or whatever they prefer and we can answer them accordingly.”’ (Educator 1, discussion session)

## Mobile devices as a positive learning method

Educators who participated in this study were very enthusiastic about the use of mobile devices to enhance teaching and learning: ‘So, the device can actually become like a preceptor or something – shadowing us. Shadowing us; not replacing us, but bringing us closer to the student in the clinical facility’ (Educator 3, FGD); ‘Because your mobile [*phone* ] is just like a further resource or even a help, if you can say it like that, an educator in … (our) absence’ (Educator 1, individual interview).

In a study by Ramos (2008:25) that deals with the use of mobile devices for learning, students express their excitement about m-learning interventions. M-learning and Internet access provide educators with an ‘open-door policy’ to learning resources from any time zone and any place to apply to their teaching practices (Ally 2009:1).

### Students’ need for practical guidance

It was emphasised by the educators that students needed guidance whilst in clinical practice: ‘I think we need to give them a guideline to develop the case study or the scenario based on that. Our students do still need guidance’ (Educator 1, discussion session).

One educator described how a student who was registered for a midwifery semester module called a clinical facilitator after midnight whilst working nightshift to ask for advice, and the patient’s life was saved because of this availability:

‘She phoned me. It was past 12:00 and we saved that life of that patient that night, because we’ve identified what was wrong with the patient. She could have given me the clinical picture and I assisted her. So our students, they does [*sic* ] need assistance. I know it’s very rare that we won’t find a registered nurse with a student, but it was a very unfortunate case, but they do need assistance out of normal clinical hours.’ (Educator 4, FGD)

Effective learning depends on the abilities of educators to encourage and support collaborative learning (Makoe 2012:94).Affordability and accessibility to m-learning could afford educators a platform to encourage and support collaborative student learning.

### Concerns about Internet access

A concern was raised about implementation of a learning intervention using mobile devices in clinical facilities, namely the need for reliable Internet access at such facilities. A need for Internet access might present a challenge to implementation of the learning intervention, since it seemed that there were participants who did not have Internet access at clinical facilities:

‘No, currently we haven’t got access to Internet, but if the students have access to [*the* ] Internet and I have access to [*the* ] Internet, then we can use this as an advantage for ourselves. But at the moment, we haven’t got Internet.’ (Educator 1, individual interview)

In an investigation into the pedagogical suitability of using cell phones to enhance learning, Makoe (2010:99) finds that use of social media applications such as Mxit and now WhatsApp allows lecturers to communicate with students and send information at an affordable cost.

### Mobile devices and ethical concerns at clinical facilities

Educators raised the fact that ethical concerns might arise as a result of use of mobile devices at clinical facilities. It was identified that ethical issues might arise when students took pictures of patients, since nurses had to be aware of confidentiality in relation to patient records. The researcher recognised that the mobile device policy of facilities needed to be implemented and formal permission had to be obtained before any information on the patient might be included in any study:

‘I think ethical issues will come in where the students have to take pictures of the patients and not because you can use the patient and take out the name to ensure privacy and confidentiality of the patient. So, when it comes to pictures, I think the ethical clearance needs to get from the administration of the facility.’ (Educator 3, discussion session)

‘I’d just like to caution the group with regard to the cell phone policies. So, we need to find out about the institution’s cell phone policy before we can continue.’ (Educator 1, discussion session)

## Ethical considerations

Ethical approval (12/10/16) for this study was obtained on 12 December 2012 from the Senate Higher Degrees Committee of the Faculty of the Community Health and Sciences, the Registrar and the Director of the School of Nursing at the University where the study was implemented. At an information session prior to implementation voluntary, informed, written, consent was obtained from prospective participants, prior to their involvement in the research project, after the purpose of the study and the expectations of the researcher had been explained. Every participant received a hard copy of the participant information form and the consent form. The participant information form explained the purpose of the study and provided guidelines to participate in the study, an outline of ethical considerations and contact details of the researcher, research supervisor and dean of the faculty should there be any questions later. Participants were informed that they could withdraw from the study at any stage. It was emphasised that there would be no negative consequence or impact on the studies of any student should they decide to withdraw from the study.

## Trustworthiness

Lincoln and Guba (1985), as cited in Polit and Beck (2012:583), provide four criteria – credibility, dependability, conformability and transferability – in order to develop trustworthiness in qualitative inquiry.

Credibility was conserved in this study through discussion during the engagement with students and educators during FGDs and individual interviews. The steps of descriptive data analysis according to Tesch (1990) were used to identify the major themes of the study.

Dependability was ensured through a rich description of the research methodology and data analysis and inclusion of direct quotations from students and educators from the FGDs, individual interviews and the discussion session held with educators.

Conformability was ensured through prolonged engagement with participants during FGDs, individual interviews and the discussion session held with educators. Transferability was ensured through inclusion of direct quotations from students and educators from the FGDs, individual interviews and the discussion session held with educators.

## Limitations of the study

Effective implementation of m-learning with the use of mobile devices in the undergraduate programme may enhance learning, but it may come up against challenges such as accessibility, connectivity, infrastructure and technical support (Traxler 2007:4).

## Recommendations

A literature review by Raman (2015:665) indicates that although incorporation of mobile technology within nursing curricula has been studied globally, a diminutive amount of literature exists to address the concerns discovered. There is a need for reflective practice with regard to the various concerns identified about the use of mobile technology, especially within environments with increasing budgetary constraints within clinical facilities.

## Conclusion

The enthusiasm that students and educators displayed about integration of mobile devices into an undergraduate nursing programme for enhancing integration of theory and clinical practice in the PHC module was promising for the possible success of such an intervention. Participants in this study identified the most suitable applications to assist this integrative process as email and WhatsApp.

Throughout the deliberations the majority of student participants indicated that their first preference to use to communicate was email, followed by WhatsApp and then Facebook to enhance integration of theory and clinical practice by incorporating m-learning into an undergraduate nursing programme at an HEI in the Western Cape Province. Information received during data collection indicated that there would be a need for training to ensure that all participants in the study were informed about how to use and thus maximise those modes of communication.

Educators would need to receive instruction about how to engage with the communication tools of choice, with the aim of gaining a deeper understanding of the potential application of these tools and their abilities to enable learning, choice, creativity, and self-direction for students. Retrospectively, students should receive explicit objectives for the use of the method of communication, as well as instruction and guidance about how they could best make use of these technologies (Ivala & Gachago 2012:162).
